# Hemodynamically significant ductus arteriosus:a new targeted approach

**DOI:** 10.1186/1824-7288-41-S1-A2

**Published:** 2015-09-24

**Authors:** Rosa Maria Cerbo, Martina Borellini, Margherita Pozzi, Savina Mannarino, Mauro Stronati

**Affiliations:** 1 Neonatal Intensive Care Unit, Department of Pediatrics, IRCCS Fondazione Policlinico San Matteo, Pavia, Italy; 2 Pediatric Cardiology, Department of Pediatrics, IRCCS Fondazione Policlinico San Matteo, Pavia, Italy

## 

Debate on hemodynamically significant ductusarteriosus (hsDA) in premature infants remains unresolved.

The association between patent ductus arteriosus (PDA) and the most adverse outcomes and comorbidities in preterms (peri-intraventricular haemorrhage, necrotizing enterocolitis, chronic lung disease, pulmonary haemorrhage and mortality) has led to the integration of ductal closure into neonatal intensive care.

The recent theory that describes PDA as “an innocent bystander” is supported by the lack of evidence of significant benefits on long-term outcomes with therapeutic interventions. So, the causal relationship between PDA and comorbidities is questioned. Moreover, PDA can close spontaneously in a significant proportion of preterms[1].

Both the traditional assumption that all PDA are pathological and the most recent theory in favor of a conservative attitudeare oversimplifications[2].

The current management of PDA does not take into account the wide range of effects attributable to a ductal shunt. A more logical approach should considerthe ductus as a clinical continuum, from thephysiological PDA to the pathological hsDA. Placingthe patient in the spectrum of “ductal disease” seems to be possible through a continuous evaluation of the hemodynamic and clinical consequences of ductal patency[3].

The current definition of an hsDA is almost entirely based on size[4]. However, the magnitude of transductal shunt relates not only to the transductal diameter, but also to the pulmonary and systemic vascular resistance and to the compensatory ability of the immature myocardium[3].

New echocardiography markers have been evaluated to estimate the impact of transductal flow on both pulmonary over circulation (left atrial/aortic ratio, anterograde pulmonary artery diastolic flow) and systemic blood flow (retrograde diastolic flow in descending aorta, left ventricular output/superior vena cava flow ratio)[5].

The possibility to evaluate flows in the middle cerebral artery, renaland superior mesenteric artery with Doppler ultrasound, allows anearly and plausible detection of regional hypoperfusion due to the “ductal steal”[3].

Other technologies that enable direct measurement of tissue oxygenation, such as Near Infrared Spectroscopy (NIRS), may also be useful to unveil the hemodynamic effects of PDA[6,7].

We are becoming increasingly aware of the role of patient's characteristics (such as genetic profile[8], BNP levels[9], antioxidant status[10]) in PDA evolution.

According to the current understanding, it seems appropriate to proposea more tailored approach to the management of PDA in preterms, based on the integration between clinical and hemodynamic status.
